# Moving toward SBIRT in the US military

**DOI:** 10.1186/1940-0640-8-S1-A84

**Published:** 2013-09-04

**Authors:** Constance Weisner

**Affiliations:** 1 Division of Research, Kaiser Permanente and Department of Psychiatry University of California, San Francisco, CA, USA

## Introduction

As is the case for many sectors of society, the U.S. military has a long history of alcohol and other drug (AOD) misuse and abuse. The issue has reached crisis proportions because of psychological stress of multiple deployments and increased use of opiates to treat pain. To better understand the problem and how it can be addressed, the Institute of Medicine of the National Academy of Sciences conducted an independent inquiry to develop recommendations for changes in the way AOD problems in service members and their families are prevented, identified and treated.

## Methods

1) Review of 10 Department of Defense (DoD) surveys of active duty military personnel; 2) Review of the related literature; 3) Analysis of policies related to alcohol and drug use, prevention and treatment of DoD and each service branch (Army, Navy, Marines, and Air Force) and Department of Veterans Affairs; and 4) site visits to military bases of each service branch.

## Results

Although prescription and other drug abuse are serious problems, alcohol is the primary problem. AOD problems are treated differently from other health conditions and are addressed treated as discipline, rather than health, problems. Drug screening is the main prevention and screening measure used, and primary care is the largest missed opportunity for early identification and treatment of AOD problems. A number of barriers were identified that limit access to screening, brief intervention and referral to treatment—including access and availability, gaps in insurance coverage, stigma, fear of negative consequences, and lack of confidential services.

## Conclusion

Recommendations include: conducting routine screening and interventions for unhealthy alcohol use, with confidential options for treatment. The increased use of technology in the prevention, screening diagnosis treatment and management of AODs was recommended in order to provide confidentiality, treatments while deployed, and for those post deployment in rural areas.

